# Can mask mandates boost nature-based tourism? The role of escapism and travel anxiety

**DOI:** 10.1371/journal.pone.0280489

**Published:** 2023-02-07

**Authors:** Mario R. Paredes, Vanessa Apaolaza, Patrick Hartmann, Aitor Marcos, Jose Domingo García-Merino

**Affiliations:** 1 School of Management and Business, Universidad del Rosario, Bogotá, Colombia; 2 Faculty of Economics and Business Administration, University of the Basque Country UPV/EHU, Bilbao, Spain; St John’s University, UNITED STATES

## Abstract

Tourism in a post-pandemic era will likely be oriented toward nature because contact with nature has restorative health benefits. The purpose of this study was to analyze the antecedents of tourists’ intentions to visit nature-based resorts during a pandemic. A nationally representative sample of the Spanish population (n = 500) was recruited by an online commercial panel to test and empirically validate the proposed conceptual framework. The findings confirmed a direct relationship between negative perceptions of wearing face masks during the COVID-19 pandemic and tourists’ intentions to visit nature-based resorts. The relationship between the perceived negative effects of wearing face masks and the intention to visit nature-based resorts was positively mediated by the need for escapism. This impact was less pronounced for anxious travelers, as shown by results corroborating the moderating effect of travel anxiety. The findings of this study contribute to research on tourism crises and provide future insights into the recovery of the industry during COVID-19.

## Introduction

The COVID-19 (SARS-CoV-2) pandemic has radically affected the tourism industry, one of the hardest hit and most damaged sectors worldwide [1, 2]. Owing to the pandemic, 2020 was the worst year for revenue in the history of global tourism, with over 1 billion fewer international travelers than the previous year. By 2021, international tourism had increased by 4%; however, it was still 72% below pre-pandemic levels [3].

This study focuses on Spain because the COVID-19 pandemic has had a historically negative impact on the country’s tourism industry [4, 5], making it a good representation of the pandemic’s worldwide impact on the sector. Spain was one of the European countries most affected by the pandemic. In 2019, tourism activity in the country represented 12.8% of its gross domestic product (GDP). However, in 2020 its GDP share dropped to 5% due to the effects of the pandemic [6]. From April 2020 to March 2021, this crisis resulted in more than 800,000 job losses for the tourism industry in Spain [7]. Two factors that strengthen the significance of Spain’s experience and establish a need for the sector’s recovery are the industry’s domestic economic contribution and the country’s participation in the global tourism industry [8].

Despite the negative scenario in the tourism industry, recent academic literature and media outlets have suggested that nature tourism emerged as an option during the COVID-19 pandemic and that this form of tourism was the branch of the industry least affected by the pandemic. For instance, between 2020 and 2021, nature and rural tourism in Spain increased by 51.8% [9] and was projected to be among the major travel trends for 2022 [10]. The pandemic has caused tourists to consider the value of contact with nature when choosing a destination [11]. As tourism recovers from COVID-19, tourists may prefer nature-based attractions [12]. Tourism in a post-pandemic era will likely be oriented toward nature because contact with nature has restorative health benefits [13], and outdoor spaces, such as natural parks, reduce the chances of contagion [14]. There is also evidence of the healing benefits of natural soundscapes to improve the well-being of visitors in facing the current public challenge of the COVID-19 crisis [15]. According to their results, the interrelationships of the restorative components are moderated by the perceived stress level, which shows significant differences before and after the COVID-19 pandemic. The post-COVID-19 visitors reported a higher level of stress and that natural soundscapes had great effects on their mental restoration [15].

The COVID-19 virus is transmitted primarily through respiratory droplets and direct or indirect contact [16]. To contain the spread of the virus, governments have imposed contingency measures such as frequent handwashing, social distancing, and wearing face masks [17]. Wearing a face mask in public spaces or near other people has become mandatory in many countries. This is the case in Spain. Before the COVID-19 pandemic, masks were used mainly by healthcare workers [18]. However, due to the pandemic, wearing a face mask has become more common and occurs for longer periods [19]. Evidence exists of the effectiveness of wearing a face mask as it reduces the risk of viral transmission, thus decreasing hospitalizations and death rates [20–22]. However, wearing a mask for long periods of time is also related to perceived harmful effects [23].

Before the COVID-19 pandemic, research on wearing face masks was primarily related to specific activities, such as medical care [24]. Mask-wearing is an unexplored area of investigation in the tourism context. Recent research has focused on the effects of wearing face masks in the hospitality and tourism sectors (e.g., [25–27]) or on the pollution generated by masks being discarded by tourists [28]. Another under-investigated area of study is the relationship between human connection to nature and the perceptions and reactions of individuals to COVID-19 [29]. According to Grima et al. [30], there is scant research on the role of nature in times of crises, especially given how green spaces help improve post-disaster resilience [31]. Thus, it is crucial to address the effects of natural areas on individuals’ reactions when facing crises, since more pandemics like the COVID-19 are expected in the future. Recently, Brewster and Gourlay [25] have called for a better understanding of the effects of wearing a face mask on customer behaviors in the hospitality industry. Thus, the present study intends to answer the following research question: Can mask mandates boost nature-based tourism? To the best of our knowledge, no research has explored how the habit of wearing face masks influences tourist behavior regarding the choice of a nature-based tourist destination.

On the basis of the evolutionary link between humans and nature, Wilson [32] proposed the biophilia hypothesis, which predicts humans’ inclination toward nature. Over the past 35 years, researchers have documented the positive benefits of nature contact on physical and psychological well-being. The biophilia hypothesis is the foundation for the nature connectedness paradigm, which examines the human–nature relationship (e.g., [33–35]). Nature connectedness has been positively associated with increased self-esteem [36], positive emotions [37], lower stress and anxiety levels [38, 39], better physical health [40], and well-being [33].

Based on the nature connectedness paradigm, this research aims to contribute to the extant tourism literature by developing and empirically testing a conceptual framework to explore the impact of negative perceptions of wearing face masks during the COVID-19 pandemic on tourists’ intentions to visit nature-based resorts. The research analyzes how this relationship is mediated by the need for escapism. Moreover, our model includes the potential moderating effect of travel anxiety due to the pandemic on the relationship between negative perceptions of wearing face masks and people’s intentions to visit nature-based resorts. The findings provide important theoretical contributions and managerial implications for the recovery of the tourism industry.

The remainder of this article is structured as follows. First, we review the literature to develop hypotheses and propose a conceptual framework. Second, we present the methodology, followed by the theoretical and practical contributions. Finally, we discuss the limitations and suggestions for future research.

## Literature review

### The effect of the negative perceptions of wearing face masks on tourist’s intentions to visit nature-based resorts

Wearing a face mask for a long period has been associated with several physiological and psychological effects. Among the most common adverse physiological effects are headaches [41], poor breathing [24], dizziness [42], facial itching, scratching [19], irritation [43], and higher facial temperatures [44]. Researchers have also recognized various psychological effects of wearing face masks. Drawing from the self-determination theory, Scheid et al. [41] identified three factors influenced by wearing face masks: (i) autonomy, the ability to have free will and choice; (ii) psychological relatedness, the feeling of connecting socially with others; and (iii) competence, feelings of capability, effectiveness, and mastery over circumstances. In a systematic literature review, Bakhit et al. [23] found fear, loneliness, stigma, and lack of empathy to be negative psychological effects of wearing face masks. The discomfort of wearing face masks has negative implications for service providers in the hospitality industry, especially in nonverbal communication [27].

While the debate about the clinical implications and magnitude of these effects continues, especially for industry workers not in healthcare or a related industry [41, 45], consensus has been reached that wearing face masks for long periods produces harmful effects and discomfort, leading to potential misuse [24, 42, 46]. Owing to the coronavirus pandemic, people have to wear masks more frequently and for longer periods than they did prior to the pandemic [19]. Thus, individuals may look for ways to reduce these negative effects in their everyday experiences.

Research across several domains has recognized the positive effect of nature connectedness on the recovery of physiological and psychological distress generated by outbreaks and social or economic crises. For instance, Littman et al. [47] explored how nature contact improves psychological and physical health for military veterans with post-traumatic stress disorder. Sachdeva et al. [48] stated that nature has restorative elements after times of collective stress, such as the negative psychological and social consequences of the Great Recession in the United States. Block et al. [49] found that nature connectedness is effective for disaster recovery related to the improvement of mental health, well-being, and social connectedness. Ottosson and Grahn [50] found that a higher connection with nature leads to a better rehabilitation process after experiencing personal negative events, such as divorce or the death of a significant other. Finally, Gladkikh et al. [51] conducted a systematic literature review on refugee populations and how nature benefits emotional and physical recovery during resettlement.

Similarly, nature-based tourism has been regarded as an interesting alternative to popular vacation destinations in the era of COVID-19 because it offers an open environment where people can avoid crowded spaces, thus diminishing the risk of contagion while having an opportunity to strengthen their connectedness to nature [14]. The nature connectedness paradigm states a positive association between high exposure to nature and individuals’ well-being [33, 38]. Nisbet, Zelenski, and Murphy [52] showed that nature connectedness is associated with self-reliance, personal growth, hedonic well-being, and purpose in life. On the basis of a systematic literature review, Qiu et al. [13] developed a conceptual framework to identify the restorative elements (e.g., physical health, psychological wellness, psychosocial development, and spiritual upliftment) of nature-based tourism, implying its appropriateness to deal with the difficulties of COVID-19. Individuals need a mechanism to deal with the negative effects imposed by the COVID-19 pandemic, such as the perceived harmful effects of wearing face masks. Based on the nature connectedness paradigm, which asserts that contact with green spaces increases well-being and helps individuals deal with negative perceptions, we propose the following:

**Hypothesis 1 (H1):** Negative perceptions of wearing face masks during the COVID-19 pandemic have a significant positive effect on tourist’s intentions to visit nature-based resorts.

### The mediating role of the need for escapism

Escapism is a coping mechanism that provides individuals with an opportunity to deal with negative experiences, such as emotional stress or anxiety produced by unpleasant realities or difficult situations [53]. People engage in various activities to shift their focus away from the problems and pressures derived from social situations [54]. Since the threat of COVID-19 diminishes subjective well-being [55], individuals escape the negative effects of the pandemic and deal with emotional distress by engaging in different activities, like binge-watching [56], internet use [57], social media use [58], nostalgic media use [59], and videogame use [60].

Escapism constitutes a major motive for tourism activities, allowing individuals to escape from their daily routines or immerse themselves in new realities and experiences [61, 62]. People escape their daily routines when they engage in tourism activities [63]. Places that offer high immersion, such as natural parks or theme parks, provide escapist experiences for visitors [64]. When tourists become immersed, they escape from the reality and stress of the world. In analyzing tourism at a mountain resort, Frochot et al. [65] found that “immersion closely tied in with the feeling of getting away: the more immersed they got, the more they forgot about everyday life” (p. 87). Recently, Park et al. [66] recognized escapism as a motivation for tourists to visit a food museum, since the experience allowed them to escape from their routines and provided meaning to daily food rituals. In the COVID-19 context, Lebrun et al. [14] identified natural parks as an alternative for tourists “to escape daily life routine and to revitalize their lives” (p. 5), since visitors may encounter an immersive experience with nature while diminishing the probability of contagion.

In a post-pandemic era, people may look for tourist destinations that offer direct contact with nature rather than traditional destinations [67]. Nature-based tourism may be an escapist option to deal with the negative effects of the COVID-19 pandemic. Escapist mechanisms may help individuals deal with the negative effects generated by the coronavirus pandemic [68]. The greater the perceived harmful effects of wearing face masks, the more prone individuals may be to escape from unpleasant realities, which may, in turn, positively influence their intentions to visit nature-based resorts. Thus, we expect the following:

**Hypothesis 2 (H2):** Negative perceptions of wearing face masks have an indirect beneficial effect on tourist’s intentions to visit nature-based resorts because of the positive effect of mask-wearing on the need for escapism.

### The moderating effect of the degree of individual’s perceived travel anxiety

Concern for safety has been a major factor in determining the travel intentions of individuals during the pandemic [12]; when traveling, the probability of physical distancing decreases and the likelihood of contagion increases from exposure to crowds [69]. Previous research has established the negative influence of disease outbreaks on travel intentions [70]. Recent evidence suggests that the COVID-19 pandemic has harmed people’s mental well-being, specifically through the generation of unprecedented feelings of anxiety, stress, and depression (e.g., [71]). These effects are magnified by the spread of information through mass media and social media [72].

Higher levels of anxiety are positively related to safety and preventive behaviors [73]. People seek to reduce anxiety by restoring feelings of control through coping strategies [74]. Wearing a face mask may serve as a self-protective behavior that diminishes an individual’s anxiety [75, 76], as well as its improperly use could increase an individual’s anxiety [77]. People may become more nervous or anxious when traveling. Anxiety and positive arousal are common feelings when traveling, generally producing minor effects on travel intentions [78]. However, the global magnitude of COVID-19 has increased risk perceptions, enlarging travel anxiety [79]. Research suggests that when perceived anxiety increases, safety perceptions decrease, negatively impacting travel intentions [80]. Recent investigations state that the coronavirus pandemic increased the travel risk perceptions of tourists, reducing their travel intentions [79, 81, 82].

Anxiety may be considered a subjective feeling experienced when individuals face a potential risk and may be influenced by variables such as personality [80], age [83], or risk aversion [84]. Risk perceptions of traveling vary among individuals. The same destination may generate anxiety for some individuals and relaxation for others [80]. People may also experience different levels of travel anxiety [78]. On the other hand, the literature has identified that nature has a positive effect on diminishing anxiety levels [85, 86]. Nature-based tourism improves the mental well-being of individuals, providing them with a mechanism of anxiety reduction [87].

Individuals who worry more about traveling because of COVID-19 experience less safety perception or feel less safe to travel and are less willing to engage in travel decisions. We expect that people with higher (lower) levels of travel anxiety, who experience greater (lesser) feelings of risk when traveling, may be less (more) susceptible to the effect of negative perceptions of wearing face masks on tourist’s intentions to visit nature-based resorts through the need for escapism and thus we propose the following:

**Hypothesis 3 (H3):** The indirect effect of the negative perceptions of wearing face masks on tourist’s intentions to visit nature-based resorts through the need for escapism is moderated negatively by individual’s degree of perceived travel anxiety due to the COVID-19 pandemic.

**Fig 1:**
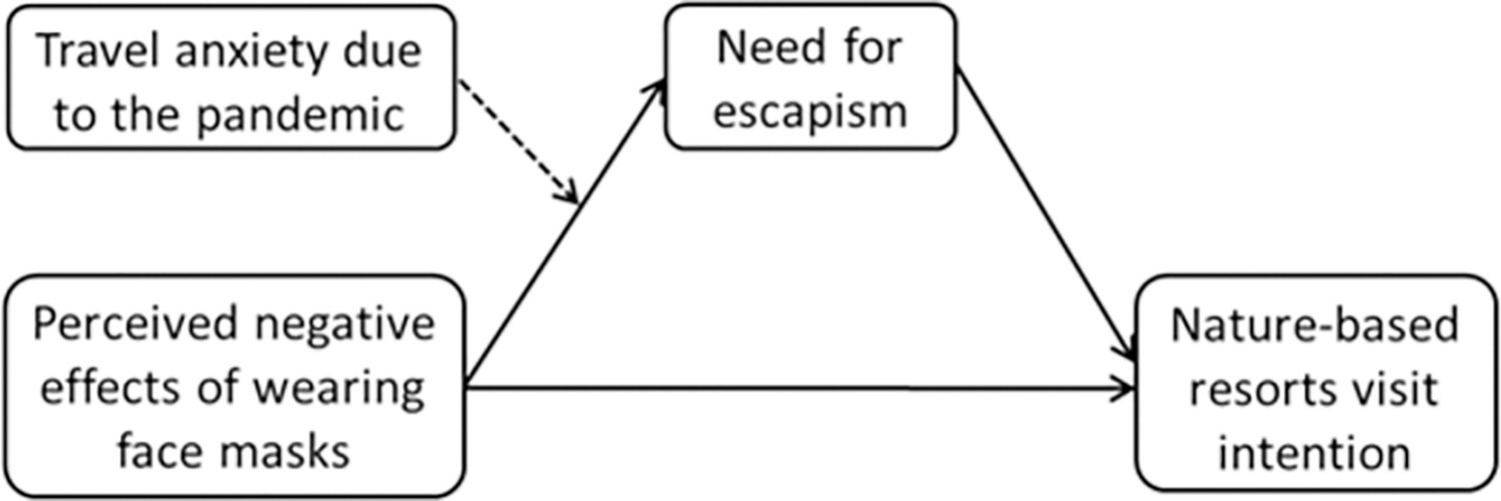
Theoretical model of the indirect effect of the perceived harmful effects of wearing face masks on tourists’ intentions to visit nature-based resorts through the perceived need for escapism.

Fig 1 depicts the theoretical model and hypothesized relationships.

## Methodology

### Data collection: Participants and procedure

To test the proposed conceptual framework, we collected data using an online survey of Spanish tourists. The sample was drawn from a nationally representative online panel of the Spanish population recruited by a commercial panel provider (n = 500, 50% female, M_age_ = 48.24, SD = 16.26, ages ranging from 20 to 84). We collected 500 responses because the required sample size to detect mediated effects above 0.14 (i.e., even small effects) with power = .80 and 0.05 is at least 462 subjects [88]. Data were collected during the first week of December 2021. The research ethics committee of the Universidad del Rosario approved this investigation under the resolution DVO005 410-CS266. All participants provided written informed consent.

Subjects were selected using a quotaـbased random sampling method from the panel until the quotas for age, gender, residential area, and income were completed (see Table 1 for details). The quotas were set to match the population approximately in socio-demographic descriptors. As a requirement, given the fact that living in an urban environment, compared to a rural environment, determines the use of a face mask to protect against COVID-19, people who lived in towns with more than 20,000 inhabitants were targets of the study. To ensure that all respondents met this requirement, a filter question was introduced at the beginning of the questionnaire which asked the participants whether their usual place of residence was in an urban center. Participants responded to the online questionnaire, which assessed to what extent wearing a face mask as protection against COVID-19 in their day-to-day lives generated negative feelings, the frequency with which they felt the need for escapism throughout 2021, and their intention to visit a nature-based tourism destination, specifically whether they had plans to visit a nature-based resort in the near future. Finally, the questionnaire contained a question to measure the level of the participants’ travel anxiety due to the COVID-19 pandemic.

**Table 1:** Sample characteristics.

			Frequency	Percentage
Gender		Male	250	50.0
		Female	250	50.0
Age		20–24	53	10.6
		25–34	69	13.8
		35–44	97	19.4
		45–54	93	18.6
		55–64	75	15.0
		> 65	113	22.6
Household income		< 600 €	32	6.4
		601–1,000 €	21	4.2
		1,001–1,500 €	53	10.6
		1,501–2,000 €	67	13.4
		2,000–2,500 €	59	11.8
		2,501–3,000 €	48	9.6
		3,001–3,500 €	31	6.2
		3,501–4,000 €	31	6.2
		4,001–5,000 €	27	5.4
		5,001–8000 €	10	2.0
		> 8,000 €	5	1.0
		I prefer not to say	116	23.2
Habitat		20,000–99,999	202	40.4
		100,000–499,999	198	39.6
		> 500,000	100	20.0
Region of residence		Andalucía	92	18.4
		Aragón	12	2.4
		Principado de Asturias	18	3.6
		Illes Balears	6	1.2
		Canarias	22	4.4
		Cantabria	6	1.2
		Castilla y León	23	4.6
		Castilla-La Mancha	23	4.6
		Catalunya	87	17.4
		Comunitat Valenciana	56	11.2
		Extremadura	14	2.8
		Galicia	27	5.4
		Madrid	66	13.2
		Murcia	8	1.6
		Navarra	3	0.6
		País Vasco	37	7.4
		**Total**	**500**	**100**

### Measurement

Validated measures in the literature were used for each of the analyzed variables. To assess the perceived negative or annoying effects of wearing a face mask as protection against COVID-19 among the participants, we used five items adapted from scales in previous studies [24, 41, 42]. Participants rated each item on a seven-point Likert-type scale (1 = *not at all*; 7 = *very much*).

Escapism was measured with a five-item scale adopted from Hirschman [53] and Wu and Holsapple [54]. Participants were asked how often they had felt the urge to run away from problems and pressures in the past year (Table 2). They rated each item on a seven-point Likert-type scale (1 = *not at all*; 7 = *very much*).

**Table 2:** Variables and measurement items.

	Mean	SD	α
***Negative perceptions of wearing face masks***	3.69	1.96	.87
Discomfort Psychological stress Skin irritation Poor breathing Dizziness			
***Need for escapism***	4.65	2.04	.93
Escape from the world of reality Escape from problems and pressures Escape from things that are unpleasant and worrisome Escape from the daily routine of life Desire to go elsewhere away from everyday activities			
***Travel anxiety due to the pandemic***	4.68	1.88	.89
COVID-19 makes me worry a lot about my normal ways of traveling. It makes me uncomfortable to think about COVID-19 while planning my vacation. I am afraid to risk my life when I travel because of COVID-19. When watching news about COVID-19, I become nervous or anxious in regard to travel. I do not feel safe to travel due to COVID-19.			

To assess tourists’ behavioral intentions to visit a nature-based resort, we used a single-item scale adopted from Han and Hyun [89]. Participants were asked about their degree of agreement or disagreement with the following statement: “I have plans to spend a few days off at a nature-based resort in the near future.” We again used a seven-point Likert-type scale (1 = *strongly disagree*; 7 = *strongly agree*).

Finally, five items adopted from the Pandemic (COVID-19) Anxiety Travel Scale (PATS) recently developed by Zenker et al. [78] were used to measure the participants’ travel anxiety. Participants rated each item on a seven-point Likert-type scale (1 = *strongly disagree*; 7 = *strongly agree*).

All measurement items and their properties are displayed in Table 2. Cronbach’s alpha confirmed the reliability of all scales.

### Data analysis

The data were analyzed with the Statistical Package for the Social Sciences (SPSS) Version 26. Mediation analyses were performed as outlined by Hayes [90] to examine whether the need for escapism mediated the relationship between the perceived harmful effects of wearing face masks during the COVID-19 pandemic and tourists’ intentions to visit nature-based resorts. The mediation analysis established whether the effect of the predictor variable (negative perceptions of wearing face masks) on the outcome variable (tourists’ intentions to visit nature-based resorts) could be explained at least partially by an increase in the perceived need for escapism, that is, whether the greater the perception of negative sensations derived from wearing masks led individuals to perceive higher levels of need for escapism and whether it is this effect that in turn increases tourists’ intentions to visit nature-based tourism destinations, such as nature-based resorts. The indirect regression coefficient was computed with a mediated regression analysis using Hayes’s PROCESS SPSS macro [90].

Next, as a first step recommended by Hayes [90], the interaction of the perceived adverse effects of wearing face masks and travel anxiety moderator on the need for escapism mediator was analyzed by moderated regression analysis to test whether the moderation was significant. To analyze the moderation of the indirect effect of negative perceptions of wearing face masks on tourists’ intentions to visit nature-based resorts through the mediator perceived need for escapism, a moderated mediation analysis was conducted using the PROCESS SPSS macro [90]. To test the significance of the moderation of indirect effects, PROCESS computed an index of moderated mediation and provided bias-corrected bootstrap confidence intervals (CI) of the coefficient. We estimated the bias-corrected bootstrap CIs with 10,000 bootstrap samples. The bootstrap interval is indicated in the Results section below, which shows the lower and upper limits of the bootstrap CI (Boot LLCI, Boot ULCI). The significance of the moderated mediation index is confirmed when the corresponding bootstrap CI does not contain zero (Bootstrap significance tests do not rely on p-values and are considered more robust than other theory tests).

## Results

The correlation analysis (Table 3) confirmed a significant positive relationship between the perceived damaging effects of wearing face masks and the need for escapism (r = .432, p < .01) and between the need for escapism and tourists’ intentions to visit nature-based resorts (r = .260, p < .01). The results also confirmed a significant positive relationship between the perceived harmful effects of wearing face masks and tourists’ intentions to visit nature-based resorts (r = .125, p < .01), providing support for H1. An analysis of indirect effects with 10,000 bootstrap samples and bias-corrected bootstrap CIs [90] confirmed that negative perceptions of wearing face masks on tourists’ intentions to visit nature-based resorts were mediated by the perceived need for escapism (b_ind_ = .134, Boot standard error [SE] = .03, 95% Boot CI [.078, .195]), providing support for H2. The mediation was complete, with the remaining direct effect being non-significant (b = .019, SE = .059, t = .33, p = .74). Thus, when the mediating variable of the need for escapism was introduced, the direct effect of negative perceptions of wearing face masks on tourists’ intentions to visit nature-based resorts ceased to be significant.

**Table 3:** Variable correlates.

	NPM	ES	IV	TA
Negative perceptions of wearing masks (NPM)				
Need for escapism (ES)	.432**			
Intentions to visit nature-based resorts (IV)	.125**	.260**		
Travel anxiety due to the pandemic (TA)	.070	.257**	.281**	

**p < .01

**Fig 2:**
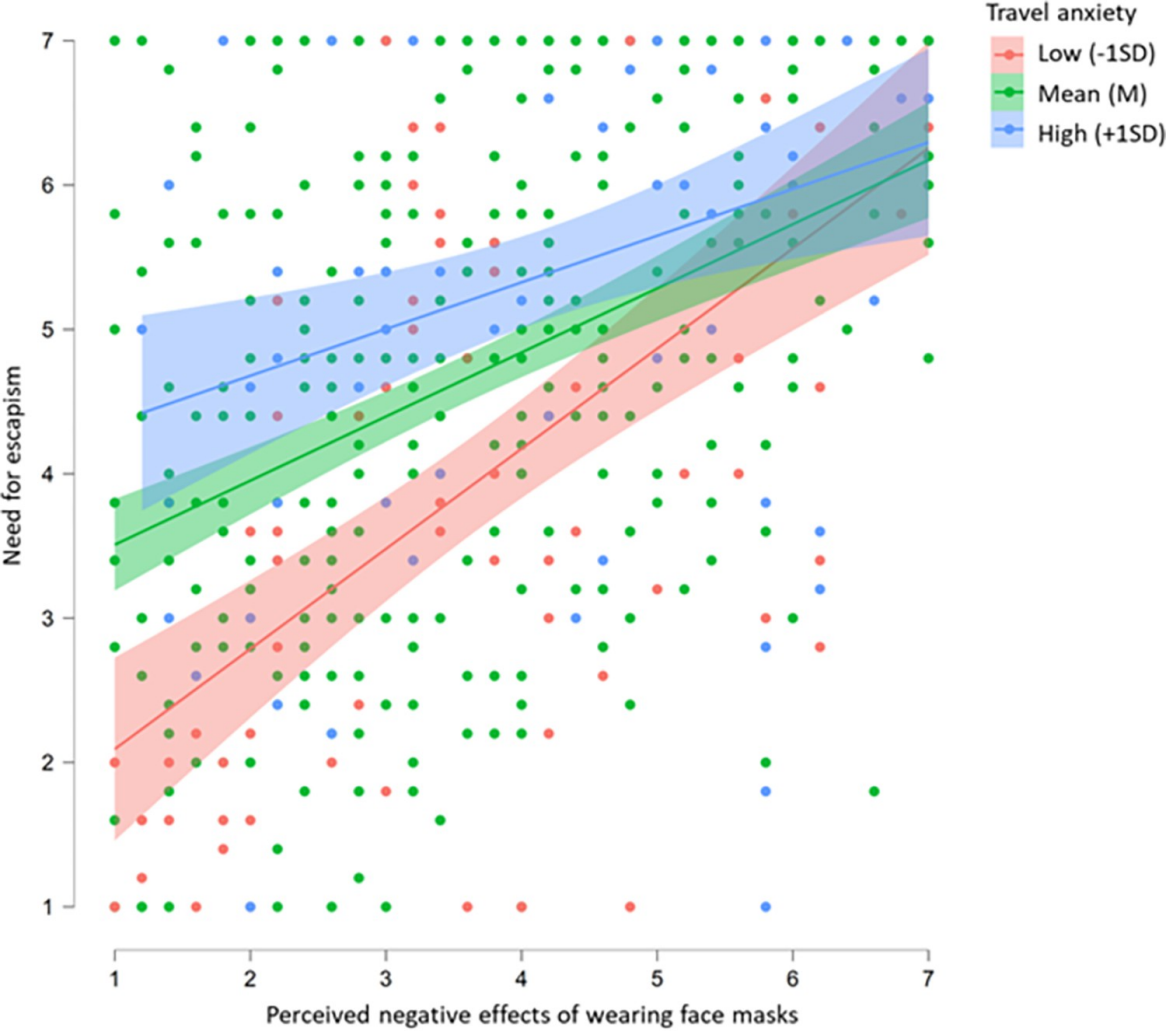
Influence of the negative perceptions of wearing face masks on the need for escapism at different levels of pandemic-induced travel anxiety.

Subsequently, using Hayes’s PROCESS [90], we tested whether the indirect effect of negative perceptions of wearing face masks on tourist’s intentions to visit nature-based resorts, mediated by the perceived need for escapism, was moderated negatively by the individual’s degree of perceived travel anxiety, as proposed in H3. The moderated mediation index that was computed supported the significance of the proposed moderated mediation (10,000 bootstrap samples) because zero was absent from the bootstrap CI (b_modmed_ = -.016, SE = .007, 95% Boot CI [-.032, -.002]). The results confirmed that the indirect effect of negative perceptions of wearing face masks on tourists’ intentions to visit nature-based resorts through a mediator-perceived need for escapism was moderated by individual’s travel anxiety levels, providing support for H3. Table 4 presents the pattern of moderation of the indirect effect of perceived adverse effects of wearing face masks on tourist’s intentions to visit nature-based resorts, mediated by the perceived need for escapism at different values of moderated travel anxiety (see also Fig 2).

**Table 4:** Moderated mediation analysis of the indirect effect of the negative perceptions of wearing face masks on tourist’s intentions to visit nature-based resorts through the perceived need for escapism.

**Index of moderated mediation**						
Mod.	Med.		Mod. med. index	Boot SE	Boot LLCI	Boot ULCI
TA	ES		-.016	.007	-.032	-.002
**Conditional indirect effect at values of the moderator**						
Mod.	Med.	Values Mod.	Cond. ind. effect	Boot SE	Boot LLCI	Boot ULCI
TA	ES	3.10 (-1SD)	.15	.03	.09	.22
		4.68 (M)	.12	.02	.07	.18
		6.26 (+1SD)	.10	.02	.05	.16

Note. Ten thousand bootstrap samples for bias-corrected 95% bootstrap confidence intervals; boot SE = bootstrap standard error, boot LLCI = bootstrap lower limit confidence interval, boot ULCI = bootstrap upper limit confidence interval; values for quantitative moderators are the mean (M) and plus/minus one SD from mean (-1SD / +1SD); Med.: mediator, Mod.: moderator, ES: need for escapism, TA: travel anxiety due to the pandemic.

Overall, the results confirmed that the positive relationship between negative perceptions of wearing face masks and tourist’s intentions to visit nature-based resorts was mediated by the level of their perceived need for escapism. Individuals who felt negative sensations or effects from wearing a mask perceived more strongly the need for escapism, which led them to develop stronger intentions to visit tourist destinations in nature, such as a nature-based resort. This relationship was negatively moderated by the level of travel anxiety experienced by individuals. As expected, tourists with average or high levels of travel anxiety due to the pandemic (in contrast to those with lower levels) were less susceptible to the positive effects of the perceived adverse effects of wearing face masks on tourist’s intentions to visit nature-based resorts, mediated by the perceived need for escapism.

## Discussion

### Theoretical contributions

The COVID-19 pandemic has dramatically transformed the tourism industry [1, 2]. This study was conducted in Spain, one of the most vulnerable economies to the pandemic [91]. On the basis of a representative sample of the Spanish population, the present research provides empirical evidence of the antecedents of people’s intention to visit nature-based resorts. Drawing from the nature connectedness paradigm (e.g., [33–35]), we developed a framework to consider the unexplored relationship between negative perceptions of wearing face masks and the intention to visit nature-based resorts, with the mediating effect of the need for escapism and the potential negative moderating effect of travel anxiety on this relationship. This work contributes to the extant hospitality and tourism literature by shedding light on unexplored areas of study that have been called for research, specifically, (i) the effects of wearing face masks on tourist’s behaviors [25] and (ii) the positive psychological effects derived from contact with nature during the COVID-19 pandemic [29]. The results of this study offer important and timely theoretical contributions to the literature.

First, our findings confirm that the intention to visit a nature-based resort is stronger for individuals with a greater perception of the negative effects of wearing face masks (e.g., discomfort, psychological stress, skin irritation). The findings imply that nature provides tourists with an opportunity to regain their sense of well-being. This is consistent with recent research highlighting the restorative effects of nature-based tourism [13] and with research on the nature connectedness paradigm, which predicts a positive effect of nature connectedness on subjective well-being [38, 52, 85]. As the pandemic continues, nature tourism offers an opportunity for people to avoid crowded spaces, thus diminishing the probability of being infected by the virus [14].

Second, our findings confirm that the need for escapism positively mediates the relationship between the perceived negative effects of wearing face masks and the intention to visit nature-based resorts. This outcome implies that individuals who have stronger perceptions of the negative effects of wearing a mask greatly recognize the need for escapism, leading them to develop the intention to visit nature-based resorts to a greater extent. This finding is consistent with the escapism literature, which suggests that individuals engage in some behaviors to avoid unpleasant realities [53, 54]. This is also in line with the literature establishing that tourism constitutes a major motivation for escapist experiences [64, 65]. Cohen [92] pointed out that an essential motivation for tourism is the desire to look for a “self-center” away from everyday activities. Vacationing offers a psychological escape from the daily routines of life [63]. Our results also extend recent research exploring escapism as a mechanism to cope with the adverse effects generated by the coronavirus pandemic (e.g., [68]).

Lastly, our findings support the negative moderating effect of experiencing anxiety for fear of contracting COVID-19 when traveling and being with other people. This confirms that the indirect effect of the negative perceptions of wearing face masks, mediated by the need for escapism, becomes weaker (stronger) for individuals with higher (lower) levels of travel anxiety. While previous research has focused on mask use as a self-protective behavior to reduce social anxiety (e.g., [76]), this research provides a novel contribution by studying the moderating effect of travel anxiety on the relationship between the harmful effects of mask wearing and consumer behavior intention to carry out nature tourism activities ("a moderator is a variable that alters the direction or strength of the relation between a predictor and an outcome" [93 p116]). Thus, our results suggest that higher (lower) levels of travel anxiety will diminish (increase) the probability that tourists will visit a nature-based resort, as a palliative to the need for escapism derived from the harmful effects caused by wearing a mask. This is because individuals with higher (lower) levels of travel anxiety will feel less (more) safe when traveling due to the COVID-19 pandemic.

This result is consistent with previous research that found anxiety experienced by travelers to be a barrier to their intentions to travel and their level of perceived safety [80]. Our findings also lend support to recent research on the negative effects of COVID-19 on travel intentions, as the fear of contracting the virus when leaving a familiar environment and being around others is a phobic stimulus that can cause anticipatory anxiety, which constitutes a persistent fear of encountering a health risk while traveling [79]. Recently, Zenker et al. [78] developed the Pandemic (COVID-19) Anxiety Travel Scale (PATS), which measures the effects of the pandemic on tourists’ travel behavior. Our findings provide empirical validation of the PATS in Spain. Specifically, evidence is provided of the moderating role of travel anxiety on the indirect effect of the negative perceptions of wearing face masks on people’s intentions to visit nature-based resorts through the need for escapism. This research demonstrates that the coronavirus pandemic has transformed conventional travel practices by raising concerns about possible infections, and people’s perceptions of health hazards, which have a negative impact on tourism.

### Managerial implications

Tourism practitioners need to comprehend the antecedents that encourage intentions to visit a nature-based resort during a pandemic. The findings of this research provide important managerial implications for the recovery of the tourism industry. First, managers who promote the benefits of nature-based resorts can profit by emphasizing the relief that a connection with nature brings in dealing with the unpleasant experience of wearing face masks on a regular basis. Hence, marketing communication should focus on highlighting the restorative benefits of nature. Tourism advertisements should highlight the safety perceptions of tourists [94]. Managers should also reassure visitors of their destinations’ safety conditions, such as uncrowded outdoor spaces where the probability of contagion is diminished, which is provided by natural places [67]. In the COVID-19 context, tourists tend to have preferences for uncrowded options [69], something that is easier to find in open, natural spaces. It is also important that service personnel in the hospitality industry continue wearing face masks because misuse may generate fear and anger in customers, thereby increasing risk perceptions [27].

Second, the need for escapism positively mediates the relationship between negative perceptions of wearing face masks and intentions to visit a nature-based resort. Nature-based resorts should position themselves as an oasis where tourists can escape from the unpleasant reality of the negative effects of the coronavirus pandemic and immerse themselves in nature and outdoor activities. These positive elements may provide tourists with memorable and restorative experiences [14]. Escapism is an important component of immersive experiences in natural parks. Escapist experiences should be highly immersive and involve active participation. They should require customers to play a key role in the overall experience [95]. Therefore, nature-based resort managers should provide various activities, such as physical or spiritual activities, that provide tourists with a complete restorative, mindful experience. This is especially important since visitors’ motivations for nature-based tourism are mainly to experience nature, keep healthy and physically fit, rest and relax in pleasant settings, improve quality of life, and pursue special interests and skills [96–98].

Finally, tourism managers should increase destination safety perceptions to reduce the travel anxiety of individuals. Recent research states that healthcare-related measures (e.g., safety protocols and hygiene habits), staff training, and the incorporation of technology to reduce contact are strategies for the hospitality industry that increase the safety perceptions of tourists [99]. If managers can effectively implement and communicate COVID-19-related practices at destinations (e.g., use of masks, well-ventilated and clean restrooms, etc.), then tourists’ anxiety will diminish, increasing their satisfaction and intentions to revisit [100]. Other mechanisms to reduce travel anxiety are providing information about the current risk level of the destination [79] and highlighting the greenness of the environment [85]. Managers should offer specific programs to strengthen nature connectedness.

### Practical implications

The findings of this study provide important practical contributions for policymakers. Research states that government measures become crucial for the recovery of the tourism industry after times of crises [101]. Owing to the magnitude of the COVID-19 pandemic, government support for the sector becomes essential [8]. Most individuals wear face masks as a consequence of government’s mandatory measures to prevent COVID-19 contagions [17]. Thus, public authorities should promote nature connectedness as an effective alternative to deal with the discomfort produced by wearing face masks. This can be done through policies that support nature-based tourism. Mechanisms such as alliances, tax reduction, or financial incentives for the sector have been proven to be effective for the improvement of the industry [102].

Governments should implement initiatives to reduce tourist’s feelings of anxiety to restore their confidence to travel. Policymakers should guarantee that the management of natural-based resorts properly follows hygiene measures. Safety protocols are one of the most effective mechanisms to prevent infections [103]. These protocols have to be updated gradually according to the evolution of the outbreak [104]. Effective communication of the measures adopted to warrant safety is also crucial to recovering tourist’s confidence [105]. Public authorities should implement training measures for hotel’s management team to properly address the tourist safety challenges generated by the pandemic [104].

Lastly, policymakers should promote communication and awareness initiatives that involve tourists and employees to comply with safety protocols. Communication should also highlight the value of nature connectedness to deal with the negative effects of the pandemic. Moreover, governments should promote mechanisms to deal with detrimental emotions produced by the outbreak. For instance, there is evidence that digital mindfulness-based interventions help individuals deal with emotional distress [106].

## Limitations and future research

This study has limitations that must be acknowledged. First, our model investigated tourists’ behaviors based on self-reported intentions to visit nature-based resorts, which may be subject to bias. Second, the magnitude, vaccination progress, health policies of the coronavirus pandemic and compliance with mask mandates vary worldwide, which probably influence frequency of mask use. Recommendations or dispositions for wearing face masks also vary across countries [17, 27]. Thus, future research could replicate these results with representative samples from other nations and cultures. Our findings can also be expanded conducting subgroup analysis that account for the frequency of mask use and other heterogeneous impacts of health policies. Finally, different types of face masks may have diverse effects on the comfort and health of individuals [107]. For instance, Luximon et al. [44] found that N95 masks increased skin temperature more than surgical masks did. Future research could consider the particular effects of different types of face masks, as well as different reasons to wear them (e.g., allergic reasons not related to the COVID-19), across the variables of the model in this study.
